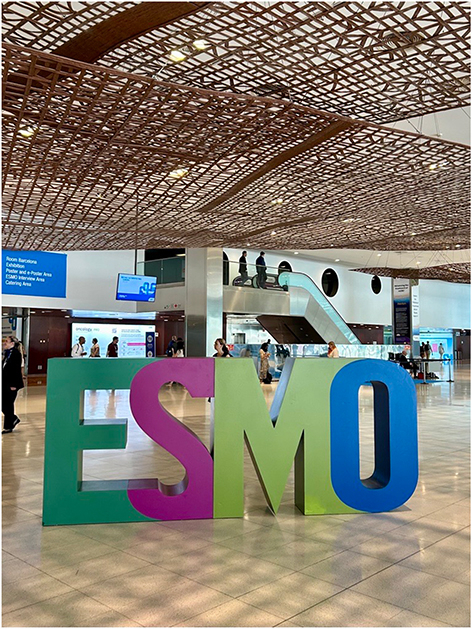# European Society for Medical Oncology (ESMO) Gastrointestinal Cancers congress 2025

**DOI:** 10.1016/j.lanepe.2025.101400

**Published:** 2025-07-17

**Authors:** Ivana Nedic

The European Society for Medical Oncology (ESMO) Congress on Gastrointestinal Cancers took place in Barcelona from 2 to 5 July 2025. Over four days, the meeting featured 34 scientific sessions and more than 90 expert speakers, showcasing the latest advances in the prevention, diagnosis, and treatment of gastrointestinal malignancies. Ivana Nedic brings together key highlights from the congress.

## Durvalumab plus FLOT for resectable gastric/gastroesophageal junction adenocarcinoma: the MATTERHORN trial

Dr Salah-Eddin Al-Batran (Frankfurt am Main, Germany) and colleagues presented results from the double-blind, phase III MATTERHORN trial (NCT04592913), which evaluated perioperative durvalumab plus FLOT (5-fluorouracil, leucovorin, oxaliplatin, and docetaxel) versus FLOT alone in patients with resectable gastric or gastroesophageal junction adenocarcinoma. Among 948 patients, durvalumab with FLOT significantly improved event-free survival (EFS) (HR 0.71, 95% CI 0.58–0.86; p < 0.001), with median EFS not reached vs 32.8 months for FLOT alone. Median overall survival also favoured durvalumab (not reached vs 47.2 months; HR 0.78), although statistical significance was not met. Patient-reported quality of life scores were similar between arms, and Grade 3/4 adverse events occurred in around 72% of patients in both groups. These results support further consideration of durvalumab plus FLOT as a potential new standard of care.

## Tumour Treating Fields plus gemcitabine/nab-paclitaxel for locally advanced pancreatic adenocarcinoma: the PANOVA-3 trial

Dr Teresa Macarulla Mercade (Barcelona, Spain) and colleagues presented results from the phase III PANOVA-3 trial (NCT03377491), which assessed Tumour Treating Fields (TTFields) combined with gemcitabine and nab-paclitaxel (GnP) vs GnP alone in patients with unresectable locally advanced pancreatic adenocarcinoma. The addition of TTFields significantly improved pain-free survival (median 15.2 vs 9.1 months; HR 0.74; p = 0.027) and overall survival (median 16.2 vs 14.2 months; HR 0.82; p = 0.039). Quality of life outcomes showed prolonged time to deterioration in pain, pancreatic pain, and global health status, alongside delayed use of pain medications and opioids. Most digestive symptom scores also favoured the TTFields group. These findings suggest that TTFields combined with GnP may offer benefits in symptom control and survival for patients with locally advanced pancreatic adenocarcinoma.

## On-demand TACE with atezolizumab and bevacizumab for unresectable hepatocellular carcinoma: the TALENTACE trial

Dr Guohong Han (Xi'an, China) and colleagues presented findings from the phase III TALENTACE trial (NCT04712643), which evaluated on-demand transarterial chemoembolization (TACE) combined with atezolizumab and bevacizumab vs TACE alone in patients with intermediate-to-high burden, unresectable hepatocellular carcinoma (uHCC). Among 342 treatment-naïve patients, median TACE-specific progression-free survival (TACE-PFS) was significantly longer in the combination arm (11.3 vs 7.0 months; HR 0.71; p = 0.009). Progression-free survival by RECIST (Response Evaluation Criteria in Solid Tumours) version 1.1 also favoured the combination (10.3 vs 6.4 months; HR 0.64; p < 0.001). Overall survival data remain immature. Grade 3–5 treatment-related adverse events occurred in 63.8% of patients receiving TACE plus atezolizumab and bevacizumab, vs 42.2% with TACE alone. These results suggest that combining immunotherapy with TACE may offer a new therapeutic option for this patient population.

## Liquid biopsy-guided anti-EGFR re-treatment in chemorefractory metastatic colorectal cancer: the PARERE trial

Dr Paolo Ciracì (Pisa, Italy) and colleagues presented results from the phase II PARERE trial (NCT04787341), which assessed sequencing of panitumumab (an anti–epidermal growth factor receptor [EGFR] antibody) and regorafenib in patients with chemorefractory, RAS and BRAF wild-type metastatic colorectal cancer (mCRC) who had previously benefited from anti-EGFR therapy. Patients without detectable RAS or BRAF V600E mutations in circulating tumour DNA (ctDNA) were randomised to receive either panitumumab followed by regorafenib or the reverse sequence. In the ctDNA wild-type group (n = 213), panitumumab led to higher objective response rates (16% vs 2%), better disease control, and longer progression-free survival (PFS1: 4.1 vs 2.4 months; PFS2: 3.9 vs 2.7 months). Benefits were more pronounced in patients with longer intervals since last anti-EGFR therapy and those without resistance mutations. These findings support the role of ctDNA-guided re-treatment with anti-EGFR agents in selected patients with mCRC.

**Figure:**